# Analysis of Transposable Elements in the Genome of *Asparagus officinalis* from High Coverage Sequence Data

**DOI:** 10.1371/journal.pone.0097189

**Published:** 2014-05-08

**Authors:** Shu-Fen Li, Wu-Jun Gao, Xin-Peng Zhao, Tian-Yu Dong, Chuan-Liang Deng, Long-Dou Lu

**Affiliations:** 1 College of Life Sciences, Henan Normal University, Xinxiang, Henan, China; 2 Key Laboratory for Microorganisms and Functional Molecules, University of Henan Province, Xinxiang, China; Leibniz-Institute of Plant Genetics and Crop Plant Research (IPK), Germany

## Abstract

*Asparagus officinalis* is an economically and nutritionally important vegetable crop that is widely cultivated and is used as a model dioecious species to study plant sex determination and sex chromosome evolution. To improve our understanding of its genome composition, especially with respect to transposable elements (TEs), which make up the majority of the genome, we performed Illumina HiSeq2000 sequencing of both male and female asparagus genomes followed by bioinformatics analysis. We generated 17 Gb of sequence (12×coverage) and assembled them into 163,406 scaffolds with a total cumulated length of 400 Mbp, which represent about 30% of asparagus genome. Overall, TEs masked about 53% of the *A. officinalis* assembly. Majority of the identified TEs belonged to LTR retrotransposons, which constitute about 28% of genomic DNA, with Ty1/*copia* elements being more diverse and accumulated to higher copy numbers than Ty3/*gypsy*. Compared with LTR retrotransposons, non-LTR retrotransposons and DNA transposons were relatively rare. In addition, comparison of the abundance of the TE groups between male and female genomes showed that the overall TE composition was highly similar, with only slight differences in the abundance of several TE groups, which is consistent with the relatively recent origin of asparagus sex chromosomes. This study greatly improves our knowledge of the repetitive sequence construction of asparagus, which facilitates the identification of TEs responsible for the early evolution of plant sex chromosomes and is helpful for further studies on this dioecious plant.

## Introduction

The origin and evolution of sexuality in dioecious plants is one of the most fascinating topics in plant biology [1]. Compared with animal systems, plants with separate sexes (dioecy) have evolved their sex systems much more recently. The evolution time of human sex chromosomes is about 240–300 million years, whereas the emergence of plant sex chromosomes arose many times during flowering plant evolution. All of these events occurred within the past 25 million years [2]. Studying such species with ‘young’ sex chromosomes can help us understand the first steps in sex chromosome evolution [3], [4].

Molecular organization and cytogenetic mapping of ribosomal genes and other repetitive DNA sequences have provided important contributions to the characterization of biodiversity. A substantial fraction of any eukaryotic genome consists of repetitive DNA sequences, including satellites, rDNA, and transposable elements (TEs). Eukaryotic genomes contain a large portion of transposable elements that have an important function for genome evolution [5]. In fact, TEs and other repetitive sequences may also have an important function in sex chromosome evolution. In the model proposed by Charlesworth et al. [6] and Rice [7], the principal step leading to the emergence and evolution of sex chromosomes (XY or ZW) is the establishment of a small sex-determining region in ordinary autosomes in hermaphrodite ancestors. The initially so-called proto-sex chromosomes are finally evolved to XY or ZW chromosomes probably via heterochromatization by the accumulation of TEs and other repetitive sequences [8], [9]. Furthermore, repetitive sequences promote chromosome rearrangements, which probably contribute to the degeneration of Y chromosomes [3].

Garden asparagus (*Asparagus officinalis*) is an economically and nutritionally important vegetable crop cultivated worldwide and belongs to the genus *Asparagus*. It has a haploid genome size of 1,308 Mb and 2*n* = 2*x* = 20 chromosomes [10], [11]. Reports have shown that sex is determined in asparagus by its sex chromosomes, where the male is the heterogametic sex (XY) and the female is the homogametic sex (XX) [12]. However, no size difference exists between the X and Y chromosomes (e.g., homomorphic). Another distinct feature of asparagus is that the YY genotype (‘supermale’) is still viable, which indicates that asparagus sex chromosomes are in an early stage of sex chromosome evolution [13]. Therefore, asparagus is a suitable dioecious plant to study the early steps of sex chromosome evolution. However, only two studies to date have explored repetitive DNA content in asparagus. Vitte et al. [14] annotated and analyzed four asparagus bacterial artificial chromosomes (BACs) from three genomic regions, with particular focus on the characterization of long terminal repeat (LTR) retrotransposons. The results revealed that LTR retrotransposons are the major components of the asparagus genome, and the elements are mostly intact, young, and nested. Furthermore, they found that several families have become particularly abundant. Hertweck (2013) sequenced, assembled, and annotated the transposable elements of 11 exemplar plants of Asparagales, including asparagus. In that study, genome coverage was limited to only 0.13× per species [15]. Detailed sequence and quantitative information about the global repeat composition of the asparagus genome are lacking, including comparisons between male and female genomes. For further studies on this dioecious plant and on understanding the evolution of plant sex chromosomes, it would be helpful to have detailed transposon-related sequence information of asparagus that has sex chromosomes at a very early stage of chromosome evolution.

High throughput, next generation sequencing (NGS) technologies that are based on fast and cost-efficient parallel processing of millions of templates, offer unique opportunities for whole-genome analysis, including not just genes but also repetitive fraction of genomes [16]. NGS technologies have been successfully used for the investigation of repetitive elements in several plant genomes [17], [18], [19]. To provide genome information for comprehensive efforts to study *A. officinalis*, we carried out a global analysis of the TE fraction of the *A. officinalis* genome via the NGS technology combined with bioinformatics approaches without a physical map of the genome. The results demonstrated that the *A. officinalis* genome could be assembled and annotated using solely next-generation DNA sequencing technologies without a full reference genome. The data obtained here might contribute to the investigation of early stages of sex chromosome evolution and lay a foundation for further studies on this dioecious species.

## Materials and Methods

### Plant Material and DNA Extraction

Seeds of *A. officinalis* variety ‘UC309’ were germinated and grown in the garden field of Henan Normal University until flower development to distinguish male and female individuals. Total genomic DNA was extracted from the leaves of one male and one female plant as described by Doyle and Doyle [20].

### DNA Library Preparation and Illumina Sequencing of Male and Female Libraries

The genomic DNA from male and female line of *A. officinalis* was sheared to an average fragments size of about 300 bp in length to construct two shotgun and paired-end libraries. The DNA-seq libraries were constructed according to Illumina manufacturer’s instructions for 101-bp pair-end library and sequenced on the Illumina HiSeq 2000 system. The raw sequence reads were quality filtered using the following criteria and a custom script: (1) filter reads including adapter sequencing; (2) discarding reads for which Ns comprised more than 3% of the total length; and (3) if a read includes low-quality bases that comprise more than the 15% of total reads, then the read was discarded. Quality control checks on raw sequence data coming from high-throughput sequencing pipelines were performed using FastQC, which could give a quick overview of sequencing quality. The sequencing data has been deposited to the Sequence Read Archive under study accession number SRP036876.

### Genome Sequence Assembly of *A. officinalis* Sequences

The pair-end reads from male and female libraries were merged and subjected to genome assembly. SOAPdenovo [21] was used for contig assembly and scaffolding with k-mer size of 23 bp. DNA paired-end reads were used for bridging scaffold gaps by using GapCloser [21].

### Validating the Accuracy of Assembly by Mapping Known ESTs and Contigs to Scaffold Sequences

To compare our assembled scaffolds with known EST transcripts, we used available ESTs from NCBI. For *A. officinalis*, 8,430 EST sequences were collected from NCBI (http://www.ncbi.nlm.nih.gov/nucest?term=Asparagus *officinalis)*. All EST sequences of *A. officinalis* were formatted and the 3′ polyA tails were trimmed using our custom perl script. These sequences were then aligned onto the scaffold sequences by using GMAP [22] to derive spliced alignments. Moreover, we aligned known contigs assembled by Hertweck [15] to our scaffolds sequence by using the BLAT software by applying the -tileSize = 18 parameter.

### Identification and Characterization of Transposable Elements

Scaffolds longer than 200 bp were used for further TE identification by similarity searches against plant repeat databases. The repeat sequences were identified using RepeatMasker (version 3.3.0, www.repeatmasker.org) with the RMBlast (version 2.2.27) search engine from NCBI to search against the RepBase library (version 17.11) [23] and the TIGR plant repeat database [24]. The TEs from assembly scaffold sequences were then categorized and annotated according to the TIGR plant repeat databases. TEs include RNA-mediated retrotransposons, DNA-mediated transposons, and miniature inverted-repeat transposable elements (MITEs) [24], [25] based on structure and sequence composition [24]. Retrotransposons consist of long terminal repeat (LTR) and non-LTR retrotransposons. LTR retrotransposons are further classified into Ty1/*copia* and Ty3/*gypsy* subclasses. Non-LTR retrotransposons include LINEs (long interspersed repetitive elements) and SINEs (short interspersed repetitive elements) [26], [27]. The percentage (% of genome) of each subclass was calculated via reads that could be mapped to portions of scaffolds that are annotated as the repeat subclass, divided by the total reads mapped to scaffolds.

### Comparison of TE Abundance between Male and Female Genomes

We compared the TE sequences between male and female genomes via the following approach: first, we mapped the reads from male and female libraries by using Bowtie [28] with the option “–a –v 0”to the assembled scaffold sequences. Second, we discarded reads that are mapped to multiple locations. We then finally calculated the read counts of each scaffold and normalized to reads per kilobase of scaffolds per million (RPKM) for comparisons. Furthermore, to identify TEs with abundance that differed between male and female genomes, we defined male-biased (female-biased) scaffolds by the following cutoff: scaffolds with RPKM (reads per kilobase of scaffolds per million) >3 in the male (female) library and RPKM = 0 in the female (male) library. The identified scaffolds were annotated to find male-biased and female-biased TEs.

## Results

### Illumina Sequencing and Assembly

To produce a scaffold-level assembly for *A. officinalis* (2n = 2x = 20), where a genome size of 1,308 Mbp (1C) was expected [10], we performed Illumina/Solexa sequencing of paired-end libraries to construct an initial *de novo* genome sequencing of *A. officinalis*, which was used as a model species for dioecious plants. A total of 50,831,394 and 35,201,336 high-quality 101 bp clean paired reads remained and resulted in 10,267,941,588 and 7,110,675,932 nucleotides for the male and female libraries, respectively. In total, 172 million reads were sequenced, which represent about 17 Gb of sequence bases and more than 12-fold coverage of the *A. officinalis* genome. The average quality score of sequencing dataset in this project is more than 36, which suggests high sequencing quantity (Figure S1).

The initial steps in *de novo* genome sequencing involved the assembly of DNA sequence reads into contiguous consensus sequences [29]. SOAPdenovo uses paired-end reads to resolve repeats, which resulted in 163,406 assembled scaffolds and ranged in sizes from 500 bp to 25,735 bp (Figure S2). The N50 size of the scaffolds, which is the length such that 50% of the assembled genome lies in blocks of the N50 size or longer [30], was 1,504 bp, and the total length of all the assembled scaffolds sequences was ∼400 Mbp, representing 30% of the estimated *A. officinalis* genome size (1,308 Mbp). Scaffolds of the *A. officinalis* genome were G-C poor (∼37%) compared with A-T content (∼63%), which was similar with the per base sequence content from raw sequencing reads (Figure S3).

To evaluate the accuracy and completeness of the genome assembly, the quality and comprehensiveness of the scaffold sequences were assessed by aligning 8,430 EST sequences to the finished assembly. More than 80% known EST sequences (7,077 EST sequences) were aligned linearly to the assembled scaffolds with identities of more than 90%.

We also compared previously published contigs with our assembled sequences. Recently, Hertweck assembled 440 scaffolds sequences of asparagus and annotated them as transposable elements sequence [15]. By comparing these non-transcribed elements with our assembled sequences, more than 87% (381/440) of Hertweck’s scaffold sequences were aligned linearly to our assembled scaffolds with identities of more than 90%. The average length for scaffold with TE elements is 735 and 1552 for Hertweck’s and our assembled sequences, respectively (Figure S4). The result from the comparison of the transcript sequences and non-transcribed elements suggested that the assembly of the *A. officinalis* genome was of high quality and coverage.

### TE Identification and Classification

**Figure 1:**
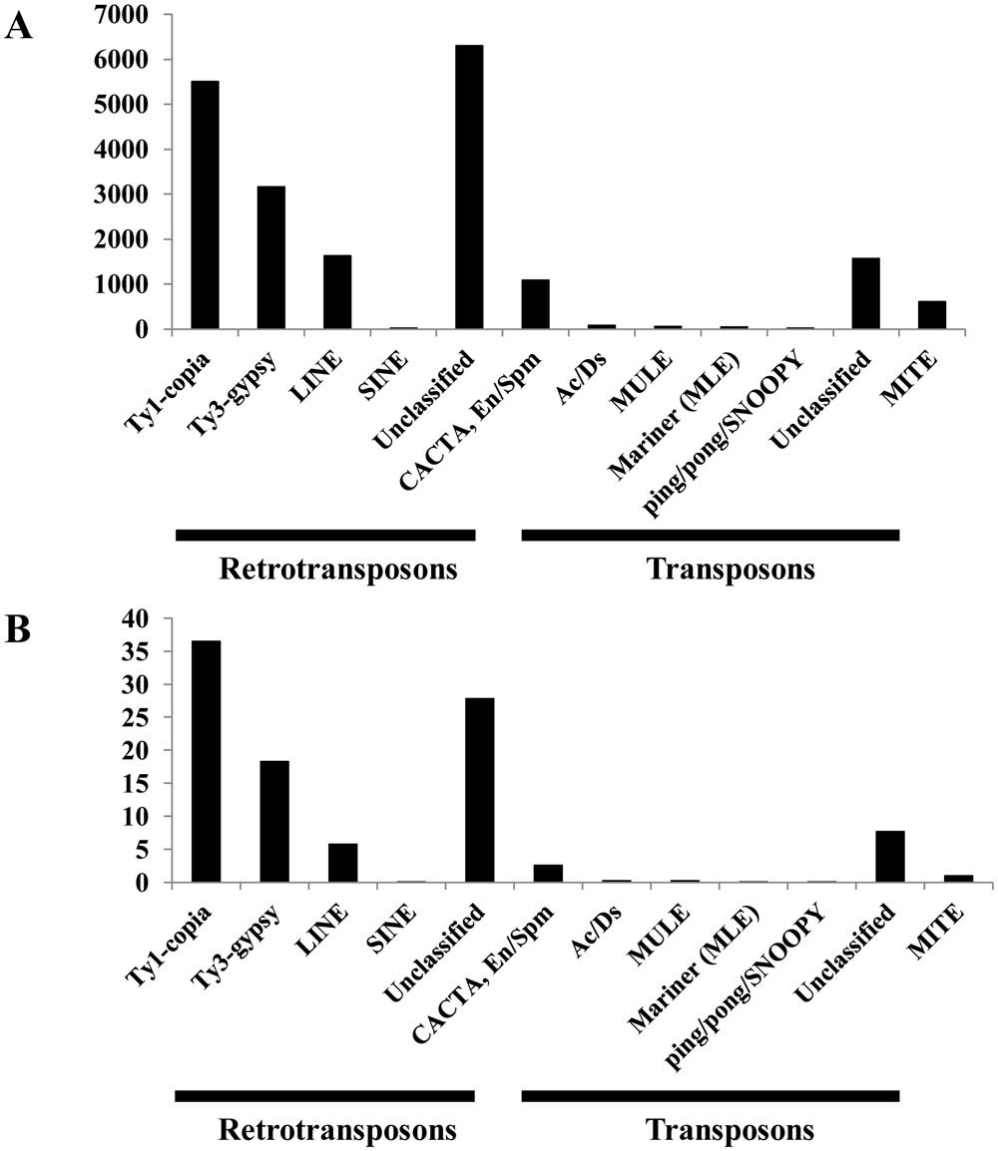
Histograms of the composition of transposable elements in *A. officinalis*. A: Number of scaffolds; B: DNA fraction (in % of the whole TE fraction).

Repetitive elements, especially transposable elements, are major components of plant genomes. However, detailed sequence and quantitative information about the global repeat composition of the *A. officinalis* genome are lacking. To understand the function of TEs on the organization and evolution of the *A. officinalis* genome, identification and annotation of TEs were performed. Overall, TEs masked about 53% of the *A. officinalis* assembly (Table 1; Figure 1).

**Table 1:** Proportion of genome containing transposable element types in *Asparagus officinalis* estimated from next generation sequencing data.

TE class	subclass	No. Scaffolds	% of TE fraction	% of genome	Genomeproportionof Male (%)	Genome proportion of Female (%)
Retrotransposons	Ty1-*copia*	5506	36.49	24.274	23.446	25.435
	Ty3-*gypsy*	3160	18.28	4.155	4.090	4.247
	LINE	1621	5.71	0.200	0.195	0.209
	SINE	23	0.03	0.009	0.009	0.009
	Unclassified	6301	27.80	19.250	18.613	20.143
Transposons	CACTA, En/Spm	1088	2.56	2.131	1.925	2.420
	Ac/Ds	78	0.21	0.007	0.00696	0.0069
	MULE	59	0.21	0.075	0.062	0.092
	Mariner (MLE)	50	0.06	0.004	0.004	0.0043
	*ping/pong/SNOOPY*	22	0.08	0.006	0.006	0.006
	Unclassified	1568	7.62	1.344	1.306	1.396
MITE	MITE	604	0.95	1.622	1.513	1.774
Total TE	–	20,080	100	53.077	–	–

**Figure 2:**
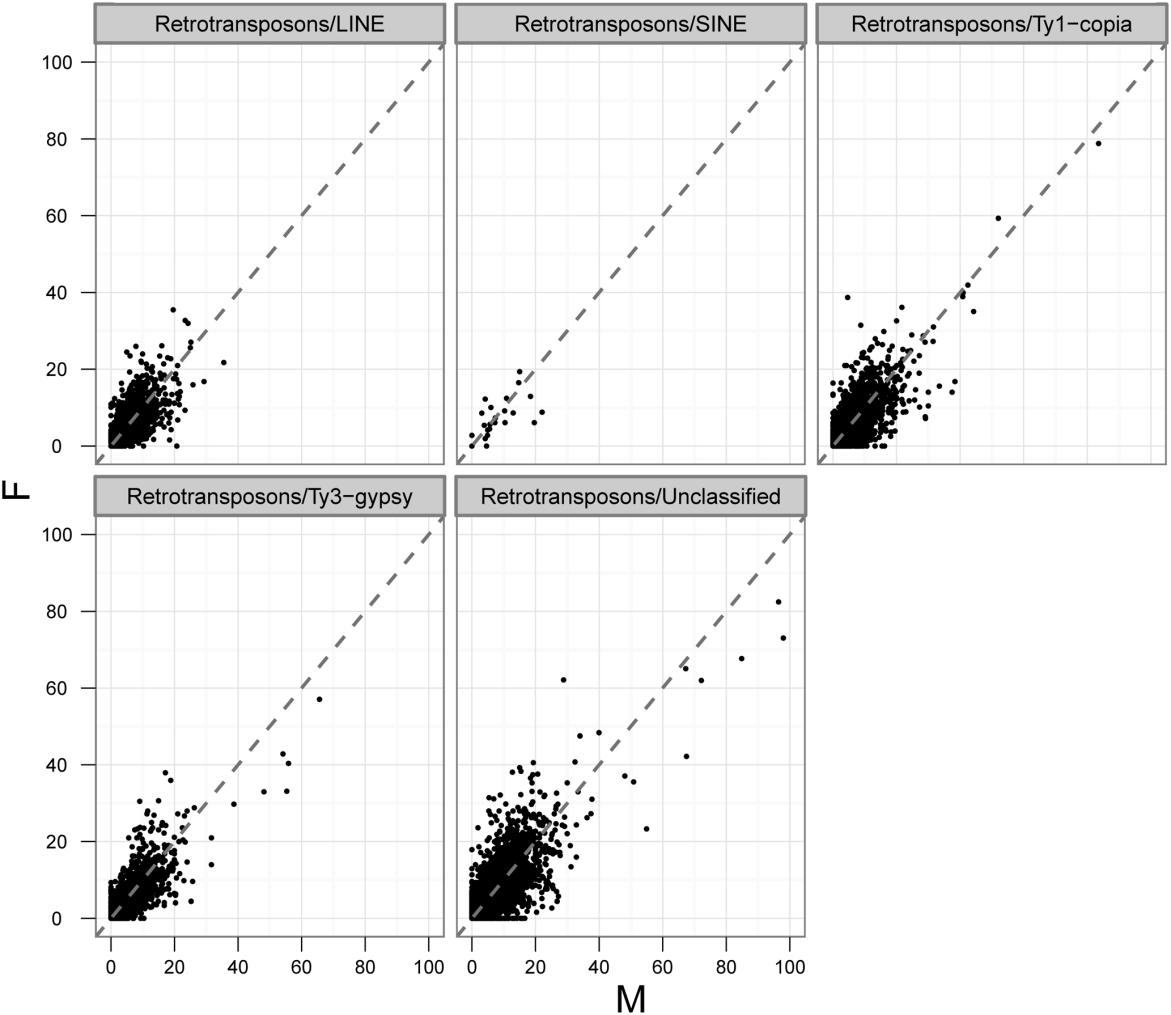
RPKM plot for retrotransposon sequences of *A. officinalis*. The plot included 5506 Ty1/*copia*, 3160 Ty3/*gypsy*, 1621 LINE and 23 SINE assembled region. X axis stands for the RPKM value for per retrotransposons sequences from male DNA-sequencing library. Y axis stands for the RPKM value for female library.

In this work, both LTR/non-LTR retrotransposons and DNA transposons were identified. The proportions of retrotransposons, DNA transposons, and MITEs were 47.9%, 3.6%, and 1.6%, respectively. Retrotransposons were more abundant than DNA transposons and MITEs. A total of 5,506, 3,160, 1,621, and 23 scaffolds were classified as Ty1/*copia*, Ty3/*gypsy*, LINE, and SINE, respectively (Figure 1, Figure 2). The most abundant DNA sequences found in the asparagus genome were LTR retrotransposons. Out of them, Ty1/*copia* and Ty3/*gypsy* represented more than 24% and 4% of the genome, respectively. Ty1/*copia*-like retrotransposons showed a relatively higher degree of diversity and was more abundant than Ty3/*gypsy*-like retrotransposons.

**Figure 3:**
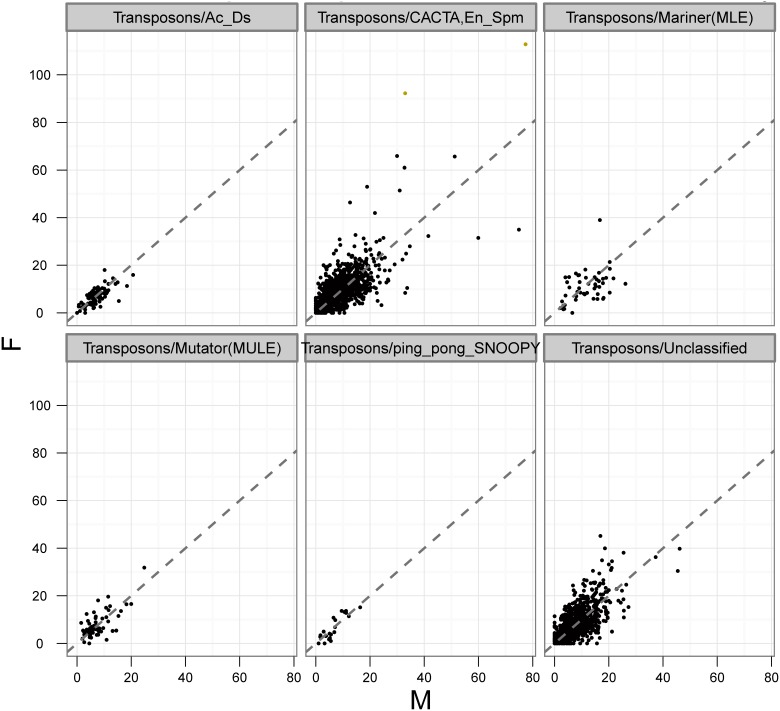
RPKM plot for transposon sequences of *A. officinalis*. There were 78 Ac/Ds; 1088 CACTA, En/Spm; 59 MULE; 50 Mariner (MLE); 22 *ping/pong/SNOOPY* sequences which were identified as transposons. X axis stand for the RPKM value for per transposons sequences from male DNA-sequencing library. Y axis stands for the RPKM value for female library.

Compared with LTR retrotransposons, non-LTR retrotransposons and DNA transposons were relatively rare. LINEs and SINEs, which belong to non-LTR retrotransposons, only made up 0.2% and 0.009% of the asparagus genome, respectively. DNA transposons, including CACTA, Ac/Ds, MULE (MUtator-Like Element), Mariner, ping/pong/SNOOPY, and other unclassified elements, accounted for about 3.6% of the genome (Figure 1, Figure 3). Within them, the CACTA subclass was the most abundant element, which involved more than half of all transposon sequences and comprised 2.1% of the asparagus genome.

### Comparison of Transposable Elements between Male and Female Asparagus Genomes

In theory, *A. officinalis* male and female genomes differ only by the constitution of their sex chromosomes; thus, the differential sequence distribution of *A. officinalis* X and Y chromosomes could be largely reflected by the differences found in repeat compositions between these genomes. Repeat quantification based on Illumina data showed that the abundance of most of the repeat groups is similar in males and females (Figure 2, Figure 3, and Table 1). However, slight differences were still observed in the abundance of several repeat groups between male and female genomes, such as subclasses Ty1/*copia*, CACTA, and MULE, which all showed higher copy numbers in female than male genomes (Table 1).

**Figure 4:**
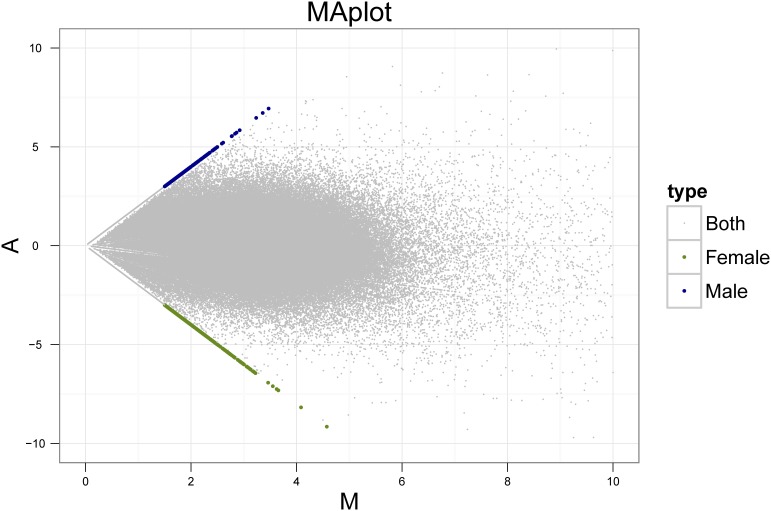
Distinctly sex-biased sequences in the genome of *Asparagus officinalis*. Let C1 and C2 denote the counts of reads mapped to a specific scaffold obtained from male and female samples, respectively. Then we define M = (C1+C2)/2, and A = C1–C2. Blue and green dots represent distinctly 248 male-biased and 553 female-biased sequences, respectively, while grey dots represent sequences which have similar abundance between male and female genomes.

Furthermore, we identified scaffolds with abundance that differed between male lines and female genomes. Although the abundance of most assembled scaffolds was similar in male and female DNA sequencing libraries, a number of scaffolds showed divergent abundance between male and female genomes. Using the methods described in materials and methods section, 248 male-biased and 553 female-biased scaffolds were detected in asparagus genomes (Figure 4). Among the male-biased scaffolds, 27 were annotated as transposable elements, including 26 retrotransposons and 1 transposon. A total of 47 scaffolds were identified as female-biased transposable elements, including 35 retrotransposons, 11 transposons, and 1 MITEs (Table S1).

## Discussion

We performed a comprehensive characterization of the TE fraction of *A. officinalis* by analyzing whole genome sequences from both male and female asparagus. The assembly process of repetitive fractions is difficult because a high sequence homogeneity among copies of a given repeat or duplication limit the potential to reconstruct sequence order [31], [32]. However, when the method described in the Material and Methods section was performed, we successfully assembly partial sequences of asparagus genome, and among which, TE sequences were detected. Of course, the assembly contigs represent only partial sequences (approximately 30%) of the asparagus genome. The detected TE sequences occupy 53% of the assembled contigs, which we interpret as the percentage of TEs of the whole asparagus genome. Our results showed that next-generation DNA sequencing technologies combined with bioinformatics analysis could provide enough information for TE characterization even in diploid organisms without prior genetic information.

In this study, transposable elements masked about 53% of the *A. officinalis* genome, which was much higher than that for a previous report on asparagus that involved the use of a very low coverage genomic sequence data (13.35%) [15]. Low coverage of the genome probably underestimated the actual repetitive fraction. Vitte et al. reported that over 72% of the three analyzed BACs of *A. officinalis* were covered by TE sequences, and two BACs out of the three were composed almost entirely of LTR retrotransposon sequences, which represent approximately 81% and 91% of the sequence analyzed, respectively [14]. Whether this phenomenon is universal or particular needs further analyses. In fact, the assembly of highly repeated sequences is the major limitation of de novo sequencing of complex genome via next generation sequencing [33]. Furthermore, TEs from asparagus are not easy to detect fully in terms of sequence identity because most non-coding regions of TEs are too divergent from the other species sequenced to be detected. Therefore, the total percentage of TEs to the scaffolds is likely underestimated in this study. In the future, this question can be solved by using constructing paired reads library with different insert lengths and deeper sequencing to cover different repeat lengths [34], so that not only the most conserved parts of TEs are detected but also structure-based less conserved regions of TEs such as LTRs.

A total of 20,080 scaffolds were identified as transposable elements, and retrotransposons were more abundant than DNA transposons. This result is consistent with the theory that retrotransposons represent one of the major forces that cause extensive genome size variation in higher plants [35], [36], [37]. Majority of identified repeats belong to LTR retrotransposons, which is similar to that found in other medium-sized plant genomes [18], [35]. Ty1/*copia* elements were present in twice as many copies as Ty3/*gypsy* and constituted an even larger portion of the genome. The prevalence of Ty1/*copia* elements over other groups of retroelements was observed in other plant genomes including banana [38], and their differential proliferation substantially contributed to the genome size variation among related species. However, in many plant species, such as rice [39], *Vicia* sp. [40], and pea [18], Ty3/*gypsy* elements were most abundant. This interesting observation indicates that particular lineages of TEs maybe specifically amplified in several species [35]. The low abundance of LINEs and DNA transposons seems to be typical for plant genomes, and similar patterns were observed in many other plants, such as in *Silene latifolia* and banana [38], [41].

The large amounts of sequencing data produced by NGS technologies not only could reveal the sequence composition of repetitive DNA, but also could be used as a tool for repeat quantification [38] and mapping and fingerprinting of other genomes. A report has shown that the genome proportions of various repeat fractions in *P. sativum* estimated via bioinformatics analysis of NGS sequencing data and their quantification via experimental methods were in good agreement [18]. To investigate the difference between male and female genome of asparagus, repeat quantification between male and female genomes were compared to reveal the differential distribution pattern of the repetitive fraction on the X and Y chromosomes. The results showed that most of the repeat groups had similar abundance in males and females, which was in agreement with the fact that the sex chromosomes of asparagus are in the early stages of sex chromosome evolution [4] and that the Y chromosomes only partially degenerated [42] and showed similar sequences with the X chromosomes. Indeed, the previous study of the model dioecious plant *Silene latifolia* also showed similar results [41].

Furthermore, we identified scaffolds with abundance that differed between male lines and female lines. Out of them, sex-specific transposable elements may be present. Repeat-induced chromatin structure changes were hypothesized to be the initial event in sex chromosome emergence [1]. Sex-specific transposable elements were useful for sex identification and may provide clues for the early steps of plant sex chromosome evolution. However, deeper sequencing is still needed to finally identify the sex-specific repetitive sequences. More useful information could be obtained if reference sequencing information is available.

In conclusion, this study represents a advance in the analysis of the nuclear genome organization in asparagus, an important vegetable and dioecious plant. The application of Illumina/Solexa sequencing provided a large amount of DNA sequence data and enabled a detailed analysis of transposon element components of its nuclear genome. Even if our sample may not be completely representative of repetitive sequences of the whole genome, we conclude that Illumina sequencing provides essential knowledge of the repetitive fraction composition and organization for the delineation of the best strategy for sequencing the entire genome. Our results showed that next-generation DNA sequencing technologies could offer enough information for transposable elements even by diploid sequence without prior genetic information. The study generated various data resources that are available for the future exploration of the asparagus genome.

## Supporting Information

Figure S1 **The distribution of mean Phred quality scores** **for** ***Asparagus officinalis*** **DNA sequencing.** ASCII character encoded the quality values for the sequences were converted into quality score which was logarithmically related to the base-calling error probabilities. X axis stands for the mean quality score which is calculated for each reads. The reads count for each mean quality score is calculated and the value is used for Y axis. Two fastq files from paired-end sequences were marked by left (/1) and right (/2), respectively.
(PDF)

Figure S2 **Plot for the length of scaffold and the count number in** ***Asparagus officinalis*** **.** X axis stands for the length of the scaffold sequence. Y axis stands for the sequence numbers for different scaffold length.
(PDF)

Figure S3 **The overall %GC of all bases in all sequences to measure the GC content across the whole length of each sequence in a file and compares it to a modelled normal distribution of GC content.** In a normal random library you would expect to see a roughly normal distribution of GC content where the central peak corresponds to the overall GC content of the underlying genome.
(PDF)

Figure S4 **Overview of the frequency distribution of the length of assembled sequences.** The length distribution of assembled sequences is plotted. A: the assembled sequences in this study; B: previously published contigs assembled by Hertweck [15].
(PDF)

Table S1 **Scaffolds annotated as male- and female-biased transposable elements in this study.**
(DOCX)
