# Supplementary figures and images for: Analysis of Transposable Elements in the Genome of Asparagus officinalis from High Coverage Sequence Data
Source: PLoS One. 2014 May 8;9(5):e97189. doi: 10.1371/journal.pone.0097189 (PMC4014616; doi:10.1371/journal.pone.0097189)

**Figure S1**

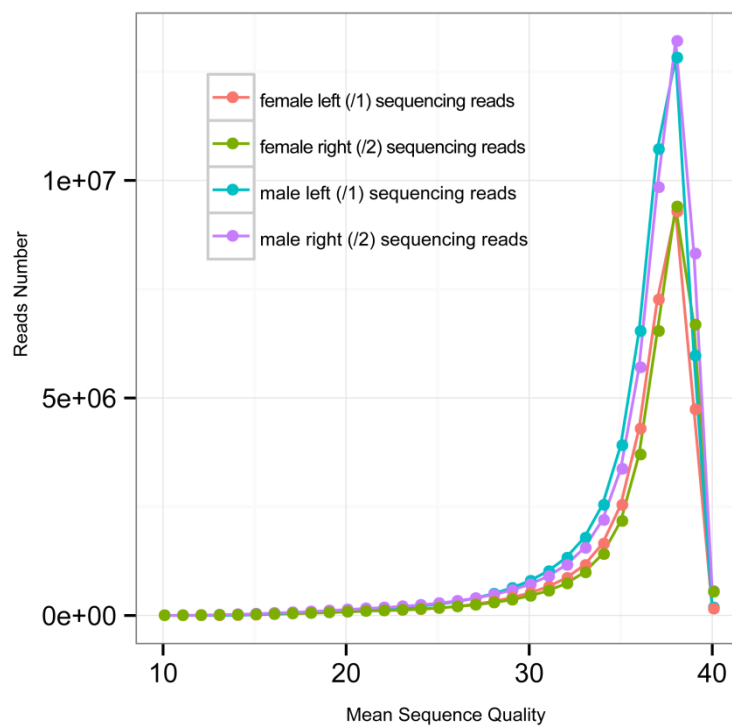

Supplement: Figure S1 — The distribution of mean Phred quality scores for Asparagus officinalis DNA sequencing. ASCII character encoded the quality values for the sequences were converted into quality score which was logarithmically related to the base-calling error probabilities. X axis stands for the mean quality score which is calculated for each reads. The reads count for each mean quality score is calculated and the value is used for Y axis. Two fastq files from paired-end sequences were marked by left (/1) and right (/2), respectively. (PDF) [file pone.0097189.s001.pdf]

**Figure S2**

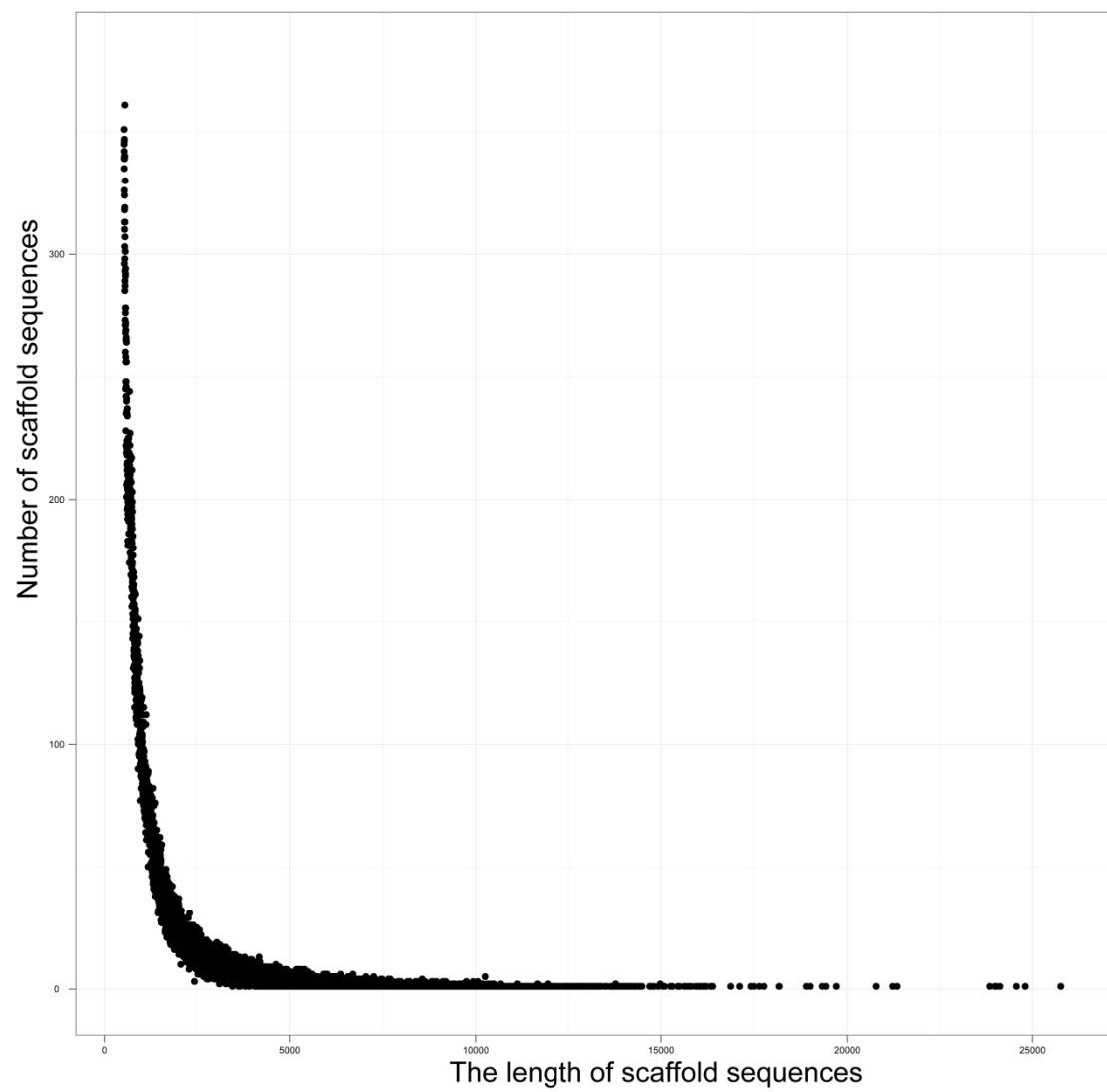

Supplement: Figure S2 — Plot for the length of scaffold and the count number in Asparagus officinalis . X axis stands for the length of the scaffold sequence. Y axis stands for the sequence numbers for different scaffold length. (PDF) [file pone.0097189.s002.pdf]

**Figure S3**

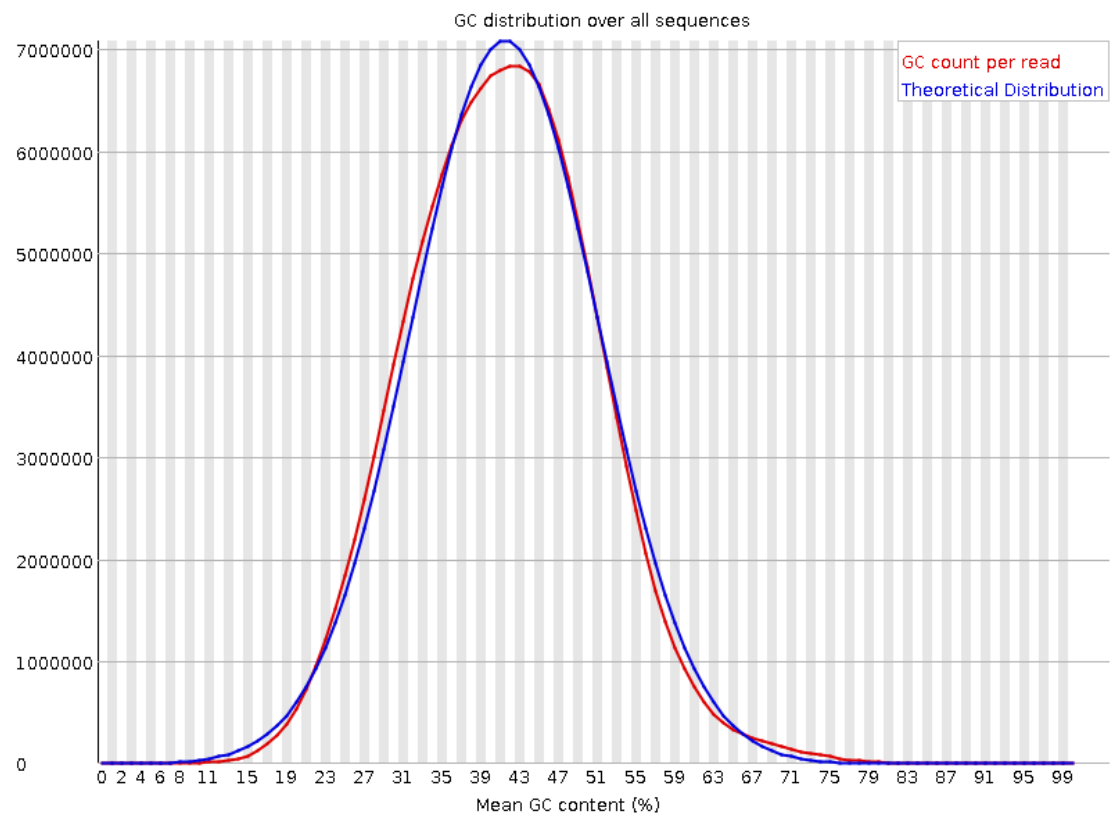

Supplement: Figure S3 — The overall %GC of all bases in all sequences to measure the GC content across the whole length of each sequence in a file and compares it to a modelled normal distribution of GC content. In a normal random library you would expect to see a roughly normal distribution of GC content where the central peak corresponds to the overall GC content of the underlying genome. (PDF) [file pone.0097189.s003.pdf]

Figure S4

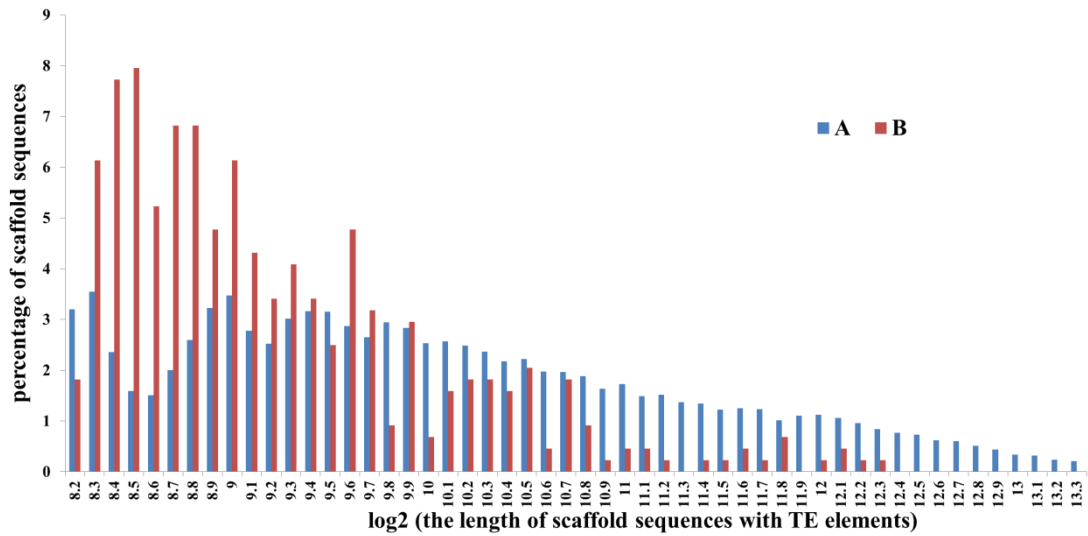

Supplement: Figure S4 — Overview of the frequency distribution of the length of assembled sequences. The length distribution of assembled sequences is plotted. A: the assembled sequences in this study; B: previously published contigs assembled by Hertweck [15]. (PDF) [file pone.0097189.s004.pdf]
