# Supplementary material for: Analysis of Transposable Elements in the Genome of Asparagus officinalis from High Coverage Sequence Data
Source: PLoS One. 2014 May 8;9(5):e97189. doi: 10.1371/journal.pone.0097189 (PMC4014616; doi:10.1371/journal.pone.0097189)
Supplement: Table S1 — Scaffolds annotated as male- and female-biased transposable elements in this study. (DOCX) [file pone.0097189.s005.docx]

Table S1 Scaffolds annotated as male- and female-biased transposable elements in this study

| Male- or female-biased | TE class | Subclass | Scaffold |
| --- | --- | --- | --- |
| Male-biased transposable elements | Retrotransposons | Ty1/*copia* | scaffold18008,scaffold4817,  scaffold22023, scaffold62257,  scaffold180491, scaffold56871,  scaffold181507, scaffold241961,  scaffold23477, scaffold142835,  scaffold223625 |
|  |  | Ty3/*gypsy* | scaffold7296, scaffold1173,  scaffold81366, scaffold53493 |
|  |  | Unclassified | scaffold11845, scaffold31320,  scaffold11364, scaffold2888,  scaffold144129, scaffold59424,  scaffold153371, scaffold126548,  scaffold97000, scaffold230867,  scaffold33244 |
|  | Transposons | Unclassified | scaffold9592 |
| Female-biased transposable elements | Retrotransposons | LINE | scaffold125181, scaffold150884,  scaffold186914 |
|  |  | Ty1/*copia* | scaffold10206, scaffold1694,  scaffold82142, scaffold16136,  scaffold3395, scaffold247323,  scaffold199624, scaffold174226,  C106763589, C106942401 |
|  |  | Ty3/*gypsy* | scaffold36, scaffold238240,  scaffold209809, scaffold5433,  scaffold4492, scaffold227733,  scaffold177425, scaffold17241,  scaffold13024 |
|  |  | Unclassified | scaffold20354, scaffold206863,  scaffold65879, scaffold69995,  scaffold246176, scaffold246008,  scaffold107083, scaffold170545,  scaffold70960, scaffold95367,  scaffold22140, scaffold144996,  C107021488 |
|  | Transposons | CACTA, En_Spm | scaffold1654, scaffold20889,  scaffold125068, scaffold263765 |
|  |  | Mariner(MLE) | scaffold10454 |
|  |  | Unclassified | scaffold20978, scaffold58710,  scaffold46163, scaffold173380,  scaffold1956, scaffold153617 |
|  | MITEs |  | scaffold249474 |
